# Development of “Advancing People of Color in Clinical Trials Now!”: Web-Based Randomized Controlled Trial Protocol

**DOI:** 10.2196/17589

**Published:** 2020-07-14

**Authors:** Alicia Chung, Azizi Seixas, Natasha Williams, Yalini Senathirajah, Rebecca Robbins, Valerie Newsome Garcia, Joseph Ravenell, Girardin Jean-Louis

**Affiliations:** 1 NYU Grossman School of Medicine New York, NY United States; 2 University of Pittsburgh Department of Biomedical Informatics Pittsburgh, PA United States; 3 Brigham and Women's Boston, MA United States; 4 Morehouse School of Medicine Atlanta, GA United States

**Keywords:** health communication, health care disparities, eHealth

## Abstract

**Background:**

Participation in clinical trials among people of color remains low, compared with white subjects. This protocol describes the development of “Advancing People of Color in Clinical Trials Now!” (ACT Now!), a culturally tailored website designed to influence clinical trial decision making among people of color.

**Objective:**

This cluster randomized study aims to test the efficacy of a culturally tailored website to increase literacy, self-efficacy, and willingness to enroll in clinical trials among people of color.

**Methods:**

ACT Now! is a randomized trial including 2 groups: (1) intervention group (n=50) with access to the culturally tailored website and (2) control group (n=50) exposed to a standard clinical recruitment website. Clinical trial literacy and willingness to enroll in a clinical trial will be measured before and after exposure to the website corresponding to their assigned group (intervention or control). Surveys will be conducted at baseline and during the 1-month postintervention and 3-month follow-up. Website architecture and wireframing will be informed by the literature and experts in the field. Statistical analysis will be conducted using a two-tailed *t* test, with 80% power, at .05 alpha level, to increase clinical trial literacy, self-efficacy, and willingness to enroll in clinical trials 3 months post intervention.

**Results:**

We will design a culturally tailored website that will provide leverage for community stakeholders to influence clinical trial literacy, self-efficacy, and willingness to enroll in clinical trials among racial and ethnic groups. ACT Now! applies a community-based participatory research approach through the use of a community steering committee (CSC). The CSC provides input during the research study conception, development, implementation, and enrollment. CSC relationships help foster trust among communities of color. ACT Now! has the potential to fill a gap in clinical trial enrollment among people of color through an accessible web-based website. This study was funded in July 2017 and obtained institutional review board approval in spring 2017. As of December 2019, we had enrolled 100 participants. Data analyses are expected to be completed by June 2020, and expected results are to be published in fall 2020.

**Conclusions:**

ACT Now! has the potential to fill an important gap in clinical trial enrollment among people of color through an accessible web-based website.

**Trial Registration:**

ClinicalTrials.gov NCT03243071; https://clinicaltrials.gov/ct2/show/NCT00102401

**International Registered Report Identifier (IRRID):**

DERR1-10.2196/17589

## Introduction

### Clinical Trials Health Disparities

Racial and ethnic minority individuals in the United States are consistently underrepresented in clinical trials [1]. Paradoxically, these groups are disproportionately affected by the leading causes of chronic disease and mortality compared with whites [2]. Participants in clinical trials are typically educated, white, and from the middle class [3,4], limiting the external generalizability to people of color (ie, African American, Hispanic, Asian, and Native American) [5,6]. Limited scientific evidence on effective solutions to address the health of racial and ethnic minority groups contributes to the health disparities gap. Identifying treatments to ameliorate the burden of disease experienced by people of color requires increased participation in clinical trials. This study will investigate the effectiveness of a culturally tailored website to increase knowledge, self-efficacy, and willingness to enroll in clinical trials among people of color in the New York City area.

### Background

Factors that may affect racial and ethnic group decision making to participate in clinical trials include the following: patient mistrust, perceived racial discrimination, transparency, awareness, culture and language, health literacy, invitation to participate in a clinical trial, social support, health insurance coverage, and pre-existing comorbidities [7-18]. Research suggests that African American and Hispanic enrollment in pharmacology clinical trials for cancer drugs and cystic fibrosis are particularly low [19,20]. Addressing the many barriers to clinical trial enrollment is critical to increasing minority enrollment in clinical trials research. We particularly aim to increase the health literacy of the many types of clinical trials that go beyond pharmacological drug testing. To that end, we aim to increase minority willingness to enroll in evidence-based interventions, including mechanistic, exploratory, pilot studies; interventional trials; and behavioral trials [21]. To engender higher decision making among people of color to enroll in clinical trials, we propose an accessible, culturally adapted, electronic health (eHealth) educational tool to enhance clinical trial literacy among racial and ethnic groups. Pew Research Center reported that 88% of blacks and Hispanics use the internet, broadly serving as an opportune channel for clinical trial health education [22]. Through focus groups among people of color, we plan to address collective concerns and lean into cultural learning and concerns about research engagement among people of color, as part of developing a representative tool for collective decision making [23]. A culturally tailored website may be an optimal strategy for influencing clinical trial decision making to enroll in clinical trials through a channel the target population is already engaged with, the internet [22]. Educational tools that address the issues and concerns related to clinical trial enrollment may aid in bridging the gap between academic research institutions and communities of color.

Health literacy is a key indicator that influences the decision to participate in clinical trials [24]. Health literacy is the ability to understand and act upon information to make recommended health decisions. Examples of proficient health literacy include understanding food labels, prescription medication use, and navigating the health care system [25]. Unfortunately, only 12% of American adults show proficiency in broad health literacy [26]. This is an important finding, as individuals with low health literacy are challenged by web-based clinical trial search engines [27]. Health literacy is a key social determinant of identifying and enrolling in clinical trials and has been associated with poor health outcomes among population subgroups, including people of color [27-29]. Given the low national health literacy rates, we decided to specifically focus on clinical trial literacy domain knowledge gains, as they predict the related outcomes of clinical trial literacy and willingness to enroll in a clinical trial.

In this study, we describe the process of developing and testing Advancing People of Color in Clinical Trials Now! (ACT Now!), a culturally tailored website to promote clinical trial participation among minority groups. Through the involvement of community members, academic researchers, and clinicians, this study will bridge the gap between groups to develop and disseminate a culturally tailored website to increase clinical trial decision making to enroll in clinical trials among people of color.

### Objective

This study will evaluate the effectiveness of a culturally tailored website to influence clinical trial decision making among people of color. Given the individual interface with the website, intrapersonal factors related to clinical trial literacy and self-efficacy were identified as predictors of how willing participants are to enroll in a clinical trial. Self-efficacy, one’s confidence in carrying out a behavior, has been consistently identified as a predictor of behavior change [30]. To this end, we identified self-efficacy and clinical trial health literacy as predictors of health behavior change. The multidisciplinary team will study the following aims:

1. To evaluate the efficacy of a culturally tailored website, compared with the New York University (NYU) Langone Health’s standard clinical trial website, using a randomized group design, to assess decision making related to willingness to enroll in clinical trials. We hypothesize that participants exposed to the culturally tailored intervention website will increase their clinical trial literacy and self-efficacy and thereby increase their decision making related to willingness to enroll in clinical trials, compared with those exposed to the standard NYU website (control).
2. To determine if intrinsic psychosocial factors (clinical trial awareness, health literacy, and self-efficacy), demographic factors (including socioeconomic, gender, and age), and medical factors (risk behavior, history of preventive behavior, and clinical diagnoses) would moderate the likelihood of enrolling in a clinical trial when exposed to the culturally tailored intervention website. We hypothesize that participants’ decision making to enroll in a clinical trial will be mediated by increased health literacy and self-efficacy, independent of their knowledge, attitudes/beliefs, intrinsic motivation, social support, and socioeconomic position.

## Methods

### Framework

Our design and testing of ACT Now! are based on the National Institutes of Health (NIH) behavior change consortium and consolidated standards of reporting trials (CONSORT) statements to inform its development [31]. Following a community-based informed approach, the guidelines of the CONSORT statements will ensure implementation fidelity and thereby enhance internal validity and generalizability. Formative evaluation, including ongoing qualitative feedback on process evaluation with the community steering committee (CSC) members and qualitative debriefing sessions for participants to provide open-ended feedback, along with quantitative surveys, will ensure that the study components are feasible and acceptable to participants and stakeholders.

### Study Design

ACT Now! uses a randomized controlled trial design to assess the following patient outcomes: (1) willingness to enroll in clinical trials and (2) behavioral intention to enroll in clinical trials. The participants’ recommendations to others about clinical trials will evaluate exposure before and after the culturally tailored clinical trial literacy intervention website. Participants will be randomized into two groups: (1) the intervention group (n=50), which will have access to the culturally tailored website in the study setting [32]; and (2) the control group (n=50), which will have access to the NYU Langone’s standard clinical trial website in the study setting [33].

At the onset of the 2-year study time frame, starting from July 2017 and ending in June 2019, refinement of study components with stakeholders, community leaders, researchers, and clinicians will be decided based on mutually agreed consensus. The formative period will allow for staff training, standardization of procedures, identification and onboarding of CSC members, and endorsement of the recruitment and implementation plan during focus groups. On development of the culturally tailored clinical trial website, participants who may be equally eligible for any NYU study will be recruited through community health fairs or the CSC member referrals. Alternate-sequencing random order will be used to randomly assign participants to the intervention or control website. The participants’ viewership and engagement with the website will be monitored using Google Analytics and Squarespace metrics. An institutional review board (IRB)–approved informed consent form was administered before study participation.

Community stakeholder partnerships will engender trust, which is necessary to ensure the implementation of our protocol and sustainability beyond study completion [34]. Through the engagement of our standing CSC led by a senior health educator, we will make inroads toward sustainable relationships for community-based research by engaging our CSC. Trusted members of the community liaise between academic and health care settings to translate concerns of the people they represent. The CSC members represent a gender-balanced, racially diverse group of people who are active on their community boards, in churches, and in the field of public health. Thus, we will employ community-based participatory research [35], an approach that works with the community as equal partners in research, to formulate project aims and develop and execute our research implementation plan.

Partnership with the CSC will be instrumental in developing a website that resonates with our target population. Quarterly meetings with the CSC are critical to website development and recruitment. CSC members will recruit participants at a neutral place of their choosing or a community site (ie, library, church, their home, or NYU Center for Healthful Behavior Change). This approach includes standardization of recruitment procedures by training CSC members and complying with a checklist of procedures during participant enrollment.

Participants will be eligible for the study based on the following criteria: self-identified as a person of color, ≥18 years old, literate, and resident in the New York area for at least three months after enrollment. On enrollment, IRB consent and baseline surveys will be completed via Qualtrics, lasting about 25 min. At baseline, participants will receive a US $25 gift card and a Wi-Fi–enabled iPad, preloaded with intervention or control materials on the browser. A research assistant will familiarize participants with the iPad, including how to access the intervention or control websites. Participants who have access to the intervention website will be granted a password-protected log-in to prevent cross-contamination exposure to both websites. After participants complete the study, voluntary debriefing sessions will be offered. They will then be scheduled for their 1-month follow-up visit, and the researchers will thank them for their time.

At the 1-month follow-up, participants will return their iPads to the NYU Langone’s Center for Healthful Behavior Change and receive US $50 as compensation for their participation and time. The participants will complete the surveys 1 month post intervention and during their 3-month follow-up.

**Figure 1:**
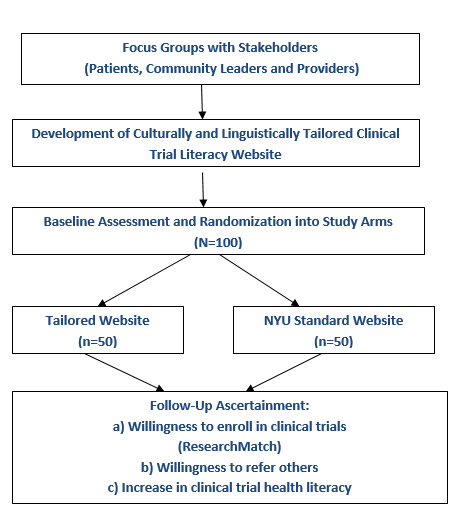
Study diagram illustrating participant flow throughout the study cycle.

During the final 3-month visit, participants will receive US $25 and complete the final surveys. Participants will have the opportunity to voluntarily debrief about their experience in the study. The research associate will thank them for their time. Participants assigned to the control arm will receive access to intervention resources no later than 3 months after their last visit. Figure 1 shows the study flowchart with the main study outcome measures.

### Ethics Approval and Consent to Participate

This study received approval from the NYU IRB in 2017. Study modifications are communicated to the IRB as modifications occur. We will communicate the IRB-approved amendments to trial participants.

### Community Steering Committee

In total, 3 CSC members will be identified by the lead health educator of the Center for Healthful Behavior Change. The CSC members will complete the collaborative institutional training initiative certification and be trained on study responsibilities and expectations. The CSC members will receive US $150 for each session, for a total of 6 meetings over the course of the 2-year study. The CSC members will participate in focus groups to aid the research team in identifying the following information: (1) beliefs and attitudes about clinical trials, (2) beliefs and attitudes about minority involvement in clinical trials, and (3) beliefs and attitudes regarding clinical trials from people in their community. They will also provide feedback on the culturally tailored website landing page, study materials, video testimonials, and the recruitment process.

### Culturally Tailored Intervention Website Development

Website design will begin with storyboarding, an outline of the digital story elements on the website [36], and information architecture, the way website content is displayed and affects user interactions with the website [37] that are informed by previous findings in the literature and expert knowledge of NYU faculty. This process will begin by reviewing data previously collected from clinical trials, which led to improved prevention strategies, treatment approaches, and recommendations for the medical management of people of color. We will identify key themes and brainstorm ideas that craft the appropriate message for the target audience at a sixth-grade reading level. This might include describing the data in the form of visual pictures, infographics on health disparities, and videos, while minimizing the use of words. We will survey web-based resources to guide the innovative creative process and development of additional content. Furthermore, we will discuss website design in an attempt to plan the layout according to how the user will navigate and process the information on the website.

After viewing the educational health materials, we will create a rough draft using concepts from the storyboard and wireframing session. We will visit this over several days before the CSC and expert panel reviews it. Finally, we will create infographics for the subheading section titled, *What is a Clinical Trial?* Video interviews with NYU faculty members of color will be included to reflect diverse representations of researchers and clinicians who could speak about the importance of including people of color in clinical trials, including benefits to the community. In addition, a video animation welcoming people to the website and providing an overview of the website will be a part of the welcome page.

Cognitive walk-through, how a person applies problem-solving techniques to learn through their interactions with a website and discover system limitations [38] and heuristic usability, evaluating the website design and literacy [39] testing will be conducted by an informatics specialist (YI). The specialist will address technical issues, layout, design, and literacy level appropriateness of the website. Consistent with previous research and testing of culturally tailored websites [40], think-aloud sessions, where participants verbalize their thought process when interacting with the website [41], will be held with 12 people reflective of the target population demographics, with a diverse age, gender, and race sample, to examine website navigation and content validation concerns [41,42]. Cognitive walk-through of study surveys allows for editing and troubleshooting corrections and time determination of approximately 25 to 30 min to complete the study.

**Figure 2:**
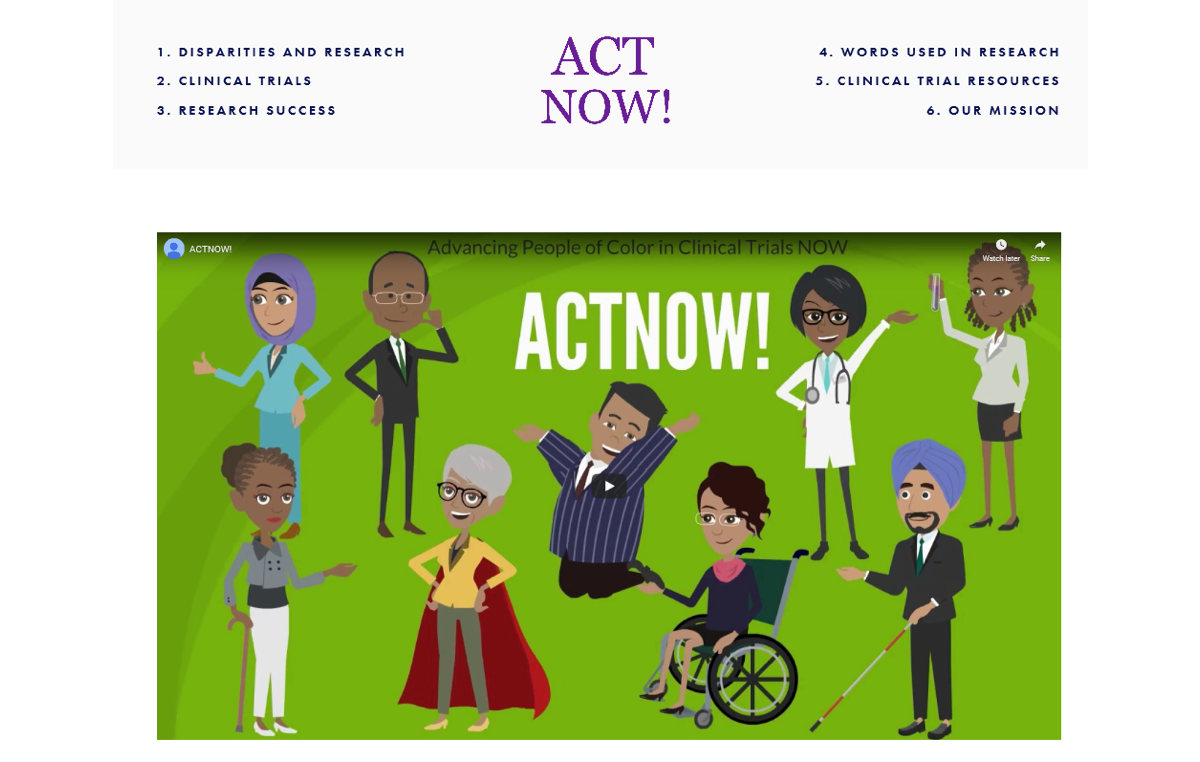
Landing page screenshot of study including subheading menus and cartoon animation describing clinical trials.

Participants enrolled in the intervention condition will be exposed to the tailored website, which will have 7 sections of content including the welcome page: *Disparities and Research*, *Clinical Trials*, *Research Success*, *Words Used in Research*, *Clinical Trial Resources*, and *Our Mission*. In addition to the *ACT Now!* landing page, each page explains the content in the video and print format. The *ACT Now!* landing page (Figure 2) includes a video animation welcoming the user to the website and providing a summary of the website content and the importance of increasing enrollment in clinical trials among racial and ethnic groups. The cartoon characters depict different ages, skin tones, cultural and religious markers (eg, the hijab), and disability status. Infographics on disparities in clinical trials and the need for minority representation in research to address chronic conditions (ie, breast cancer and diabetes), images of people of color interacting with health care professionals, and an iPad include pictures of patients and providers interacting as well as descriptive narrative content.

**Figure 3:**
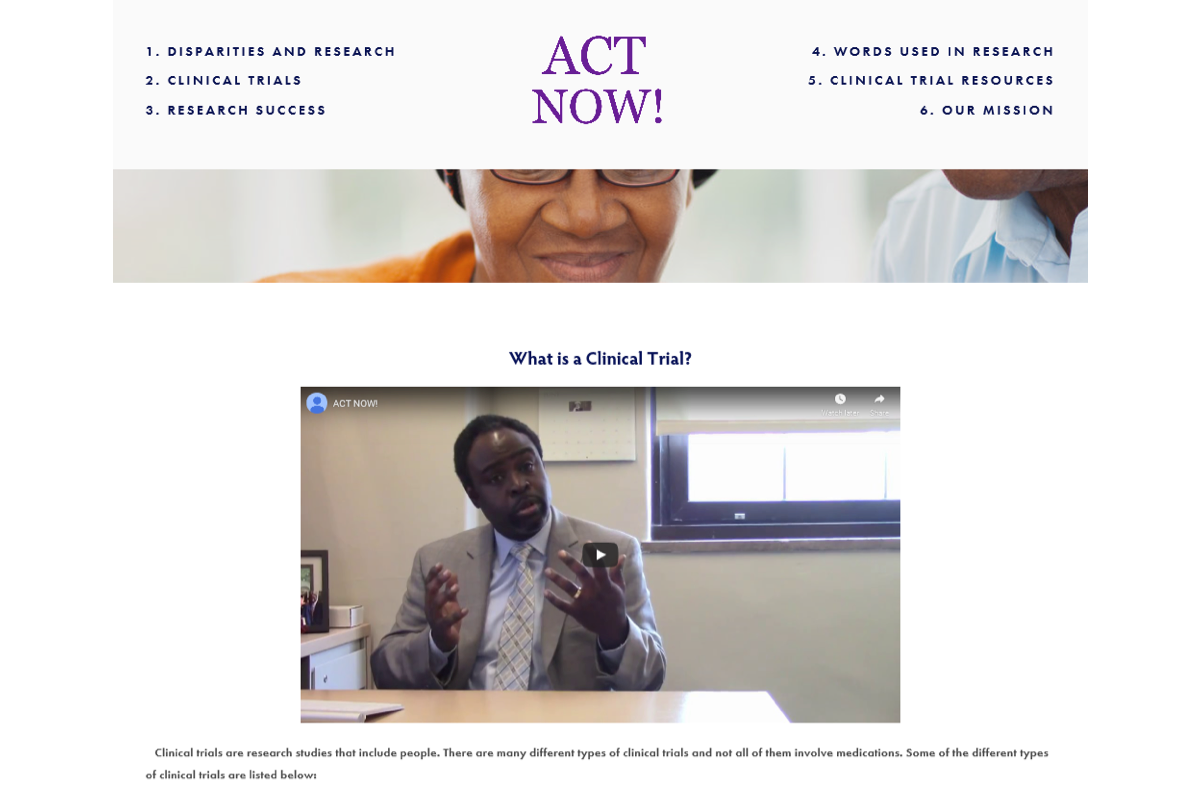
Joseph Ravenell, MD describing the different types of clinical trials, including community-based research.

The *Disparities and Research* section includes 3 paragraphs describing how social determinants such as race, ethnicity, age, education, and income affect health outcomes and how increasing diversity in research may help identify solutions to reducing health disparities. This information will explain health disparities in research by an NYU faculty member and physician embedded in video format on the website. Similarly, the *Clinical Trials* section includes the following subheadings: *What is a clinical trial?*, *What are the steps of a clinical trial?*, *Common barriers in a clinical trial?*, *Reluctance to participate in clinical trials?*, and *Importance of participation in clinical trials?* (Figure 3) Each subheading page will include 1 to 3 paragraphs on the page topic and a video of an NYU faculty member of color. Each page will have information explaining what takes place during clinical trial research and explain clinical trial involvement from the participant’s point of view. Website topics include the role of the IRB, participant time, compensation, and acknowledging historical events in America that may have led to patient mistrust, such as the Syphilis Study at Tuskegee. This section will explain that research participation is voluntary and that safeguards are in place to protect the rights of the participant.

The *Research Success* section of the website will include 3 subheadings: *How has research benefited my community?* *Areas of needed improvement*, and *Testimonials*. The *How has research benefited my community?* section will include information on the role of research in neighborhoods of people of color, such as Harlem, to provide screenings and behavioral health interventions to reduce chronic disease risk factors. This section provides information regarding successful behavioral health interventions in the community, such as barbershops, that have increased screening and treatment rates for African American men, who are typically underserved. The *Words Used in Research* section includes definitions of a glossary of terms used throughout the website. The *Clinical Trial Resources* section explains the safeguards in place to protect a person enrolled in clinical trial research, with hyperlinks to the NIH and ClinicalTrials.gov as additional sources that explain the process. The *Find a Clinical Trial* section will include hyperlinks to clinical trials at NYU and the NIH organized by the hyperlink disease state. The final *Our Mission* section will an image of the Center for Healthful Behavior Change staff, the name and contact information of the principal investigator, key faculty and staff, and acknowledgment of the funding source.

On study completion, all participants in the control arm of the study will have access to the intervention site via a partner community website.

### Measures

Screening questions will take about 5 min. Participants will complete the survey measures at baseline, 1-month follow-up, and 3-month follow-up. Baseline data collection and subsequent measures will take approximately 25 min each for eligible participants enrolled in the study. We will use Qualtrics software to capture survey data entry in real time. Secured access to participant data is only available through the NYU Langone Health password log-in–required secure portal. We will code surveys before statistical analysis using validated survey tools. The clinical trial sponsor did not deem that a data monitoring committee was necessary.

#### Sociodemographic Variables

Sociodemographic variables will include age, race, insurance status, level of education, employment status, marital status, and annual household income.

#### Health Status and Chronic Conditions

The medical outcomes study questionnaire short form 36 health survey is a validated 36-item instrument that serves as an indicator of overall health status. This survey has a Cronbach alpha reliability score of approximately .80. Estimates of reliability in the physical and mental sections are typically above 0.90. The eight sections of the tool examine vitality; physical functioning; bodily pain; general health perceptions; physical, emotional, and social role functioning; and mental health [43]. These eight sections include scaled scores that are weighted sums of questions in each section.

#### Clinical Trial Literacy

Researchers at the NYU Center for Healthful Behavior Change and the University of California, Los Angeles, developed and validated a 24-item survey instrument to assess clinical trial literacy and attitudes across a diverse sample of 400 participants. Survey items will be developed in partnership with the CSC members to help assess literacy level appropriateness. Likert scale responses ranged from definitely false to definitely true. Sample survey items include “A clinical trial is a research study that involves people.” and “Research is important to improve the health of people of color.” Item score correlations ranged from 0.45 to 0.68, and Cronbach alpha based on Pearson correlations was .93.

#### Message Effectiveness Scale

Culturally tailored messages are more effective when they resonate well with specific populations to improve targeted behavior outcomes [44]. An assessment of website content will ascertain what was more influential in increasing the willingness of the participants to enroll in a clinical trial. The 14-item message effectiveness scale [45] will be completed 1 month postintervention to assess user acceptance.

#### Internet Self-Efficacy Scale

The internet self-efficacy scale will measure the self-confidence of users in using the iPad and the internet based on a 5-point Likert scale response option. The 8-item survey reported a Cronbach alpha of .93 [46].

#### Willingness to Enroll in a Clinical Trial

The willingness of the participants to enroll in a clinical trial will be assessed based on 3 hypothetical trials on a scale from 1 (*not willing*) to 5 (*very willing*). Clinical trial vignettes are based on weight loss, hypertension treatment, and cancer treatment [47]. Cronbach alphas of .6 and .8 were reported for the Corbie-Smith distrust in clinical research index and the primary care assessment survey trust subscales, respectively. Table 1 displays an overview of the study measures at baseline, 1 month, and 3 months.

**Table 1:** Study outcome measures.

Measures	Baseline	1 month	3 months
Demographic and clinical variables	X^a^	—^b^	—
Medical outcomes study questionnaire short form 36	X	—	—
National assessment for adult literacy	X	—	—
Clinical trials literacy	X	X	X
Message effectiveness scale	X	X	X

^a^X denotes what measure to ascertain.

^b^Empty cells signify not to capture the measure at the timepoint.

### Recruitment

The CSC members will be recruited via the word-of-mouth snowball method and will occur at a place of their choosing in a neutral community site (ie, library or faith-based organization). Although effective eHealth frameworks exist for eHealth clinical trial recruitment [47], the CSC took ownership of the study recruitment process. Eligible participants enter into a research arm based on a predetermined randomization spreadsheet. Nondisclosure of group assignment during enrollment blinds trial participants to intervention arm assignment. This randomized approach includes standardizing recruitment procedures by training the CSC members and complying with a checklist of procedures during participant enrollment. The CSC members will be trained in study recruitment procedures before launching the study, including thorough role-playing of participant enrollment and troubleshooting of iPad equipment. NYU staff accompany the CSC members during participant enrollment to ensure consent forms are signed in compliance with the NYU Langone Health regulations.

### Power

Using a medium effect size (*d*=0.32) and a sample size of 100, this study will be adequately powered (>80%) to detect significant differences between participants with improvements in health literacy scores. A significant increase in the number of participants in the intervention arm who are willing to register for clinical trials can also be detected. Actual registration in a clinical trial is not measured.

### Statistical Analysis

Paired *t* tests and chi-square tests will be conducted to compare participants who exhibited improvement in health literacy compared with those who did not. A logistic regression model will determine which factors are associated with a higher likelihood of (1) enrolling in a clinical trial and (2) referring others to participate in clinical trials as well, based on baseline factors. A two-tailed test, at 80% power and an alpha of .05, comparing 25% with 45% would require 50 participants enrolled in each arm. This will allow for differences to be assessed in participation rates. In addition, a 23% attrition rate is taken into account when considering the mediating and contextual factors between tailored clinical trial messages and participation rates. The attrition rate is based on our previous study experience with a similar study design. We plan to prevent study attrition with biweekly check-ins with participants regarding iPad troubleshooting, website engagement, and/or general study questions.

Communication on trial results will be disseminated to the scientific community via a peer-reviewed publication. A CSC meeting will be held at study completion to share trial results with community stakeholders.

## Results

We will design a culturally tailored website that will provide leverage for community stakeholders to influence clinical trial literacy, self-efficacy, and willingness to enroll in clinical trials among racial and ethnic groups. ACT Now! applies a community-based participatory research approach through the use of a CSC. The CSC provides input during the research study conception, development, implementation, and enrollment. CSC relationships help foster trust among communities of color. ACT Now! has the potential to fill a gap in clinical trial enrollment among people of color through an accessible web-based website. Funded in July 2017 and obtained IRB approval in spring 2017. As of December 2019, we had enrolled 100 participants. Data analyses are expected to be completed by June 2020, and expected results are to be published in fall 2020.

## Discussion

### Principal Findings

The significance of this study is its patient-centered approach to addressing minority enrollment in clinical trials. Building the intervention website in partnership with community leaders in a way that values their perspective is an important cornerstone of our study. Eliciting community feedback throughout the process of website development and recruitment is crucial to ensure acceptability and dissemination of the message regarding the importance of participation in clinical trials. Logistical challenges with administrative onboarding of the CSC members slowed down study initiation. In addition, securing iPads with the appropriate access restrictions and image permissions and website vendors were operational hurdles. However, building inroads in the community can erect bridges where barriers once existed and allow for involvement in community-based research in the future.

Previous interventions to increase minority enrollment in clinical trials have focused on just one racial or ethnic group (ie, African American or Asian) [5,48], gender [49], or are heavily focused on drug or biologic testing (ie, cancer) [50,51]. ACT Now! uses an inclusive approach, targeting all groups of color (ie, African American, Asian American, and Hispanic). This study could be adapted by different interest groups to be used for future clinical trials for recruitment efforts, awareness platforms, and education efforts, such as the *All of Us* research program, which aims to create a diverse racial and ethnic database of patient health information [52]. In addition, this study is neither gender specific nor age specific, aiming to address women and nongender binary people of color who are typically not represented in clinical trial research as well [53]. ACT Now! is inclusive of individuals of varying racial, gender, age, and sexual orientation to reduce misconceptions and address barriers to research [7].

Engaging people of color in the study process has effectively increased knowledge about clinical trial research, such as in the Asian American community. Ma et al [5] applied the community-based participatory research approach for the development, tailoring, implementation, and evaluation of an intervention to increase Asian American representation in clinical trials. Community-based organizations and community health educators significantly increased clinical trial knowledge among 247 participants. Benefits to science and the larger Asian American community were found to be the most important factors to emphasize when aiming to enhance Asian American participation in clinical trials [5]. Similarly, ACT Now! will include people of color as part of the CSC to provide feedback on key content areas, layout, and design of the culturally tailored website, to better resonate with the target population.

ACT Now! aims to address clinical trial literacy across a diverse group of racial and ethnic minority groups, as well as self-efficacy and decision making to enroll in clinical trial research, using a CSC to guide development and recruitment. Wells et al [49] applied a cultural competency and recruitment training program (CCRTP) to increase minority enrollment in cancer clinical trials. Significant improvements were found in knowledge and attitude measures post intervention. Minority participation in clinical trials increased by 1.2% (an additional 300 minority patients) 1 year after the CCRTP. Both studies reported significant increases in knowledge gains but only modest improvements in minority enrollment in clinical trials [49].

### Study Limitations

Although our study aims to increase the literacy of all types of clinical trial research, including nonpharmacological observational studies, we do not measure actual participant enrollment in clinical trials after study intervention. Future research should consider the effect of clinical trial literacy tools to influence actual study enrollment. In addition, other barriers to enrollment should be considered, such as religion.

### Conclusions

ACT Now! will apply a community-based participatory research approach through the use of a CSC, providing input during the intervention website development and a study referral source for participant enrollment. Each CSC member will serve as a community representative that the participants trust. CSC members will ensure the safe return of iPads disseminated in the community, which sparked the recruitment referral snowball approach. In addition, NYU staff members will join CSC members at community recruitment events throughout New York City, meeting participants where they are and ensuring their onboarding process addresses any technical issues or study concerns.

ACT Now! has the potential to fill an important gap in clinical trial enrollment among people of color through an accessible web-based website. Literacy level–appropriate text, infographics, videos, and cartoon videos aim to educate people of color on clinical trial research via a web-based intervention. The culturally tailored approach, endorsed and codeveloped by trusted community leaders, strengthens the partnership between the community, academia, and the private sector.

## Abbreviations

ACT Now!: Advancing People of Color in Clinical Trials Now!
CCRTP: cultural competency and recruitment training program
CONSORT: consolidated standards of reporting trials
CSC: community steering committee
eHealth: electronic health
IRB: institutional review board
NIH: National Institutes of Health
NYU: New York University
